# Hippocampal Function Is Impaired by a Short-Term High-Fat Diet in Mice: Increased Blood–Brain Barrier Permeability and Neuroinflammation as Triggering Events

**DOI:** 10.3389/fnins.2021.734158

**Published:** 2021-11-04

**Authors:** Gabriela Cristina de Paula, Henver S. Brunetta, Daiane F. Engel, Joana M. Gaspar, Licio A. Velloso, David Engblom, Jade de Oliveira, Andreza Fabro de Bem

**Affiliations:** ^1^Postgraduate Program in Biochemistry, Department of Biochemistry, Federal University of Santa Catarina, Florianopolis, Brazil; ^2^Multicenter Graduate Program in Physiological Sciences, Federal University of Santa Catarina, Florianopolis, Brazil; ^3^School of Pharmacy, Federal University of Ouro Preto, Ouro Preto, Brazil; ^4^Laboratory of Cell Signaling, Obesity and Comorbidities Research Center, University of Campinas, Campinas, Brazil; ^5^Department of Clinical and Experimental Medicine, Faculty of Medicine and Health, Linköping University, Linköping, Sweden; ^6^Postgraduate Program in Biological Sciences: Biochemistry, Department of Biochemistry, Federal University of Rio Grande do Sul, Porto Alegre, Brazil; ^7^Department of Physiological Science, University of Brasília, Brasília, Brazil

**Keywords:** high fat diet, cognition, neuroinflammation, blood–brain barrier, memory, depression, mitochondria, bioenergetics

## Abstract

Worldwide, and especially in Western civilizations, most of the staple diets contain high amounts of fat and refined carbohydrates, leading to an increasing number of obese individuals. In addition to inducing metabolic disorders, energy dense food intake has been suggested to impair brain functions such as cognition and mood control. Here we demonstrate an impaired memory function already 3 days after the start of a high-fat diet (HFD) exposure, and depressive-like behavior, in the tail suspension test, after 5 days. These changes were followed by reduced synaptic density, changes in mitochondrial function and astrocyte activation in the hippocampus. Preceding or coinciding with the behavioral changes, we found an induction of the proinflammatory cytokines TNF-α and IL-6 and an increased permeability of the blood–brain barrier (BBB), in the hippocampus. Finally, in mice treated with a TNF-α inhibitor, the behavioral and BBB alterations caused by HFD-feeding were mitigated suggesting that inflammatory signaling was critical for the changes. In summary, our findings suggest that HFD rapidly triggers hippocampal dysfunction associated with BBB disruption and neuroinflammation, promoting a progressive breakdown of synaptic and metabolic function. In addition to elucidating the link between diet and cognitive function, our results might be relevant for the comprehension of the neurodegenerative process.

## Graphical Abstract

An illustrative scheme summarizing the main outcomes in Swiss mice fed high-fat diet (HFD), with their exact time of onset of changes in the hippocampus. An increase in the expression of proinflammatory cytokines, together with the permeability of the blood–brain barrier was detected after 2 days of HFD. Even in the first week of dietary intervention, memory and learning impairment, depressive-like behavior, and synaptic changes were observed at 3, 5, and 7 days, respectively. Later hippocampal alterations (after 4 weeks of HFD consumption) include mitochondrial dysfunction and astrocytic activation.

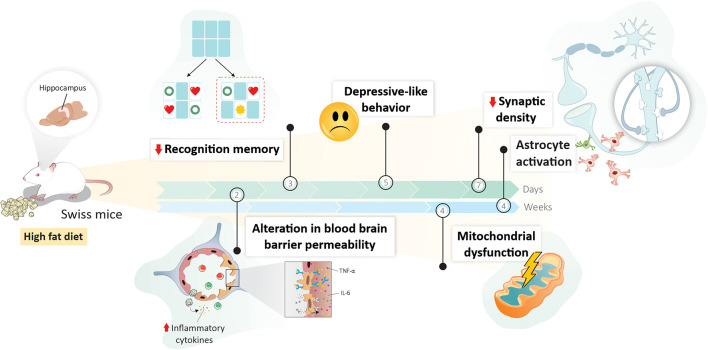

## Highlights

- A short-term HFD impacts hippocampal-dependent learning and memory, and mood behavior in Swiss mice.
- Neuroinflammation and BBB dysfunction represents a trigger events in hippocampal dysfunction mediated by HFD.
- The inhibition of TNF-α inflammatory pathway mitigate the behavioral impairments and BBB dysfunction in HFD fed Swiss mice.

## Introduction

Although we are aware that the mantra “Eat right and Exercise” is the secret to promote weight stability and good health, the obesogenic environment in Western society promotes high consumption of high-fat diets (HFDs) and sedentary behavior (Reardon et al., 2003; Asfaw, 2011). According to the World Health Organization (WHO), the prevalence of obesity has doubled in the last three decades worldwide, and currently, at least one third of adults over 20 are overweight or obese (Arroyo-Johnson and Mincey, 2016). Studies have demonstrated the adverse health consequences of energy dense diets, especially regarding disruption of the energy homeostasis, leading to chronic metabolic disorders, such as type 2 diabetes (T2D) (Freeman et al., 2014) and cardiovascular disease (Kratz et al., 2013). Equally worryingly, more recent evidence points to the impact of dietary fat on brain function and behavior (Davidson et al., 2013; Kratz et al., 2013).

A large number of studies has shown that the exposure to HFD strongly affects the hypothalamus (Velloso and Schwartz, 2011; Thaler et al., 2012). Due to its direct link to appetitive behavior and gut afferent information, the hypothalamus plays a major role in understanding the Western diet-derived metabolic changes (Velloso and Schwartz, 2011). Notably, the hippocampus may also be particularly susceptible to damage by dietary factors (Morris et al., 2006; Davidson et al., 2007; Francis and Stevenson, 2011; Kanoski and Davidson, 2011; Gibson et al., 2013; Baym et al., 2014; Hao et al., 2016; Attuquayefio et al., 2017). The hippocampus is critical for many types of learning and memory processes, and injuries/impairments in this area can be found even in early phases of neurodegenerative dementias, including vascular dementia and Alzheimer’s disease (AD) (Kanoski and Davidson, 2011). High intake of fat and sugar is associated with impairments in hippocampal-dependent learning and memory in children (Baym et al., 2014), adults (Francis and Stevenson, 2011; Gibson et al., 2013; Attuquayefio et al., 2017), and the elderly (Morris et al., 2006), suggesting a negative impact on hippocampal function across the lifespan. Similarly, preclinical investigations in rodent models have corroborated epidemiological data showing that the HFD can induce hippocampal-dependent memory impairment after a long-term of feeding (longer than 4 weeks) (Ross et al., 2009; McNay et al., 2010; Hao et al., 2016; Saiyasit et al., 2020).

In addition to the disturbances in cognitive function epidemiological data indicate that overnutrition and obesity is also related to mood disorders (Campbell and MacQueen, 2004; Toups et al., 2013; Mansur et al., 2015). This association is believed to be bidirectional, in which obese individuals tend to develop anxiety and depression and, at the same time, those who have depression end up adopting a hypercaloric diet, leading to a gradual increase in body weight (Toups et al., 2013; Mansur et al., 2015).

Another open question is how quickly a shift to HFD impacts hippocampal functions and mood-related behavior. Despite the interest and ascendancy in the number of publications evaluating the impact of HFD on the hippocampus, there are few studies that assess and highlight its consequences in a short-term (up to 4 weeks) feeding, showing only isolated effects (Gibson et al., 2013; Kaczmarczyk et al., 2013; Beilharz et al., 2014; Attuquayefio et al., 2017; Spencer et al., 2017; Wang et al., 2020).

Although many questions about the impact of HFD on hippocampal function remain unanswered, it is known that HFD consumption effects on cognitive and emotional abilities involves several independent mechanisms, including (1) inflammatory signaling such as glial cell activation and recruitment of immune cells (Pistell et al., 2010), (2) abnormalities in cellular bioenergetics, mainly mitochondrial dysfunction (Carraro et al., 2018), (3) increase in blood–brain barrier (BBB) permeability (Kanoski et al., 2010), and (4) impairment of synaptic plasticity (Hwang et al., 2010; McNay et al., 2010; Liu et al., 2015). However, the causal relationship and the time-course of these events upon a HFD routine are not yet well established. In this study, we aimed to elucidate how rapidly HFD induce hippocampal dysfunction and which mechanisms are involved in this process.

## Materials and Methods

### Animals

Six-week-old male Swiss mice were obtained from the animal facility of the Federal University of Santa Catarina, Brazil. Experimental protocols and procedures adhered regulations by the National Institute of Health Guide for the Care and Use of Laboratory Animals and were approved by the Federal University of Santa Catarina’s Ethical Review Committee for Animal Experimentation (Protocol number 6191300316). At the beginning of each experimental protocol, mice were randomly divided according to specified experimental groups and housed on grid roofs cages (4–5 per cage). They were maintained on a 12:12 h light:dark cycle with a temperature-controlled environment (22 ± 1°C) and *ad libitum* access to food and water.

### Experimental Diet and Study Design

**FIGURE 1:**
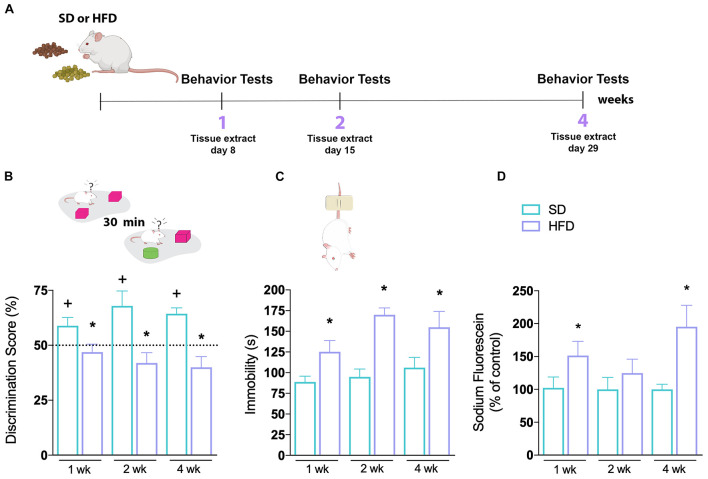
Hippocampal abnormalities of mice fed a high-fat diet. **(A)** First time-course experimental design. **(B)** Novel object recognition (NOR) and **(C)** tail suspension tests (TSTs) were performed in mice fed SD or HFD for 1, 2, and 4 weeks. Cognitive performance of SD and HFD-treated mice was accessed by NOR (*n* = 7–8/group; one-sample *t-*test with hypothetical value of 50%). Time of immobility in TST was recorded in order to evaluate depressive-like behavior (*n* = 7–8; unpaired *t*-test for 1, 2, and 4 weeks of diet, respectively). **(D)** Sodium fluorescein assay to evaluate BBB permeability on hippocampus (*n* = 5–7; *t*-test for 1, 2, and 4 weeks of diet). Values are mean ± SEM. ^+^Means higher than the hypothetical value of 50%, *p < 0.05 SD vs. HFD, ^+^discrimination score above 50%.

For all experiments, mice were randomly divided into two groups, fed either standard chow (SD), composed by 10% calories from fat, 20% from protein and 70% from carbohydrates (Nuvilab^®^ CR-1, Nuvital, Brazil), or a HFD composed by 60% calories from fat, 12% from protein and 27% from carbohydrates (PragSolutions Bioscience^®^ n-60, São Paulo, Brazil). In the first step of the experiments, mice were subdivided in other three groups and received SD or HFD for 1, 2, or 4 weeks, totalizing six different experimental groups. All the groups were followed by memory and depression-related behaviors tasks (*n* = 7–8) performed in different cohorts of animals (Figures 1A–C). At the end of the different experimental periods, mice were euthanized by exsanguination under terminal anesthesia and hippocampi were dissected for either mitochondrial function analysis (*n* = 5–6) or BBB permeability assessment (*n* = 5–7). Another cohort of animals was transcardially perfused and had their brains removed for immunofluorescence assay (*n* = 4).

In the second round of the experiments, mice were randomly selected for feeding on SD or HFD for 1 up to 6 days. Same behavioral tests were performed in different groups of animals (*n* = 6–8) followed by hippocampal dissection for BBB permeability (*n* = 4) or gene expression evaluation (*n* = 4). Another cohort of animals was transcardially perfused and had their brains removed for immunofluorescence assay (*n* = 4–5). During the last series of experiments SD or HFD-fed mice were treated concomitantly with the anti-TNFα monoclonal antibody Infliximab (10 μg/kg, intraperitoneally) once a day, for 7 days (Figure 5A). A 0.9% saline solution was given as vehicle. At the end of the treatment period, animals underwent the behavioral tests described in the previous experimental designs (*n* = 7–8), and the BBB permeability was evaluated in the hippocampi (*n* = 4). Each set of behavioral tests, as well as the *ex vivo* protocols, was performed at the same time of day, interspersing the animals and samples between groups to avoid any time bias. Body weight gain, food and water intake were evaluated in all experimental groups as described recently (Brunetta et al., 2019).

### Behavioral Tasks

#### Novel Object Recognition

All sessions were performed between 8 am and 3 pm. The task was carried out in an open field arena as described before (Cohen et al., 2015; De Paula et al., 2020) with some modifications. Briefly, after 3-day habituation sessions on the arena, animals were submitted to a 5-min-long session of training (with two identical objects) followed by the test session (with a novel object distinct in shape, color, and size) after 30 min (Figure 1B). Increased exploration of the novel object is indicative of functional recognition memory. Results were expressed as percentage of time exploring each object during the test session. A discrimination index was calculated – (Tnovel × 100)/(Tnovel + Tfamiliar), where Tnovel was the time spent by the animals exploring the novel object and Tfamiliar was the time spent by the animals exploring the known object. Exploration of object was defined as sniffing or touching the object while looking at it, or “directing the nose” toward the object at a distance less than or equal to 2 cm. In all groups, the minimum exploration time of 20 s was stipulated for inclusion in the tests.

#### Tail Suspension Test

Consists of suspending mice above the ground by their tails based on the fact that animals subjected to this short-term inescapable stress will develop an immobile posture (Figure 1C). The test is useful in assessing depressive-like behavior, given by the increased immobility time. In summary, mice were acoustically and visually isolated and suspended 50 cm above the floor by adhesive tape placed approximately 1 cm from the tail’s tip. Immobility time was recorded during a 6 min period. Mice were considered immobile only when they hung passively and completely motionless. The immobility time was recorded by a blinded observer to the experimental group (Steru et al., 1985).

### Fluorescein Assay

To assess BBB leakage, 4% sodium fluorescein (376 Da, Sigma-Aldrich Chemie GmbH, Switzerland) was diluted in 0.9% saline and injected in the dorsal penile vein (4 ml/kg) of mice anesthetized with isoflurane and allowed to circulate in the bloodstream for 30 min. Animals were then transcardially perfused with 50 ml of 0.9% saline and the hippocampi were quickly dissected and weighed. The tissues were homogenized in 7.5% trichloroacetic acid to precipitate the proteins, and centrifuged for 10 min at 10,000 × *g* at 4°C. Then, 0.1 ml of the supernatant was added to 0.25 ml (1:2.5 volume) of 1 M potassium phosphate buffer (TFK; pH 7.0). The fluorescence was scanned at an excitation and emission wavelengths of 485 nm and 538 nm, respectively, using a fluorescence Microplate Reader (Spectramax Paradigm). Data are calculated using a calibration curve of sodium fluorescein and were expressed as percentage (%) of leaking fluorescence (de Oliveira et al., 2020), compared to the SD group. Blank samples were performed to avoid artifacts.

### Immunofluorescence Staining and Image Analysis

Animals were anesthetized with tribromoethanol and perfused transcardially with saline followed by 4% paraformaldehyde (PFA) prepared in 0.1 M phosphate-buffered saline (PBS). Fixed brains were removed, embedded in 4% PFA for 24 h, and cryoprotected in a 30% sucrose solution in PBS at 4°C. The brains were then frozen and stored at −80°C for later analysis. Serial coronal sections (30 μm) of hippocampi were obtained with a cryostat (Leica) at −20°C. Groups of 6–8 sections per animal were incubated at room temperature for 2 h with 5% horse serum (HS) solution in PBS containing 2% Triton X-100. After blocking, free-floating sections were incubated overnight at 4°C with the antibody against glial fibrillary acidic protein (GFAP, mouse polyclonal, 1:400, Sigma, G3893) or synaptophysin (SYP, mouse polyclonal, 1:150, Abcam, ab8049) in 1% HS diluted in 0.5% PBS-Tx. Sections were then washed in PBS and incubated for 2 h with anti-mouse Alexa 488 (1:400, Invitrogen, A-11001) in 1% HS diluted in 0.5% PBS-Tx at room temperature. After a final washing step, sections were mounted with CC/Mount (Sigma) and covered with coverslips. Images from all 6–8 serial sections of each mouse hippocampi were acquired parallel to the coverslip (xy sections) on OLYMPUS BX41 (GFAP) and Confocal Zeiss Upright LSM780-NLO (SYP) microscopy, with a *Z*-stack reconstruction for the latter. Fluorescence was quantified on ImageJ software, evaluating the hippocampal CA1 region for SYP and the sum of the CA1, CA3, and dentate gyrus regions for GFAP.

### High-Resolution Respirometry

The Oxygraph-2k (O2k, OROBOROS Instruments, Innsbruck, Austria) was used for measurements of respiration to evaluate mitochondrial function. Hippocampus homogenates were performed in a high-potassium respiration buffer (Sims and Blass, 1986) using substrate-uncoupler-inhibitor titration (SUIT) protocols (Makrecka-Kuka et al., 2015) with modifications. Pyruvate and malate (5 and 2.5 mM, respectively) were used to determine Complex I (CI) linked LEAK respiration. ADP was added at 550 μM final concentration, which was saturating for oxygen flux to obtain OXPHOS capacity linked to CI. Succinate (5 mM) was added to reconstitute convergent CI&II-linked respiration. Titrations with the uncoupler FCCP (0.1 μM) were performed to determine the maximal electron transfer system (ETS) capacity. Rotenone (1 μM to inhibit Complex I) was added to determine phosphorylating CII (CII*p*). All experiments were performed at 37°C.

### Real-Time PCR

For the expressions of TNF-α, IL-1β, and IL-6, mRNAs were measured in the hippocampus of mice submitted to SD or HFD up to 6 days. Intron-skipping primers were obtained from Applied Biosystems. Glyceraldehyde-3-phosphate dehydrogenase (GAPDH) was used as an endogenous control for normalization of the different genes. The following primers were purchased from Applied Biosystems: GAPDH (4351309), TNF-α (Mm 99999068_m1), IL-1β (Mm00434228_m1), and IL-6 (Mm99999064_m1). The optimal concentrations of cDNA and primers, as well as the maximum efficiency of amplification, were obtained through a 5-point, twofold dilution curve analysis for each gene. For the relative quantification of genes, real-time PCR reactions were performed in triplicate from 3.0 ng of reverse-transcribed RNA, 200 nm of each specific primer, TaqMan^TM^ (Applied Biosystems), and RNase free water to a final volume of 20 μl. The relative gene expression data values were performed in an ABI Prism 7500 sequence detection system (Applied Biosystems).

### Statistics and Data Representation

Values are represented as mean ± standard error of the mean (SEM). Statistical analysis and graphs were done using the software GraphPad Prism7^®^ (GraphPad Software, La Jolla, CA, United States) or Statistic software package (StatSoft Inc., Tulsa, OK, United States). Because all data resented normal distributions, significant differences were evaluated by Student’s *t*-test and one-way or two-way analysis of variance (ANOVA), depending on the experimental design. Following significant ANOVAs, multiple comparisons were performed using Duncan’s and Dunnett’s *post hoc* test. The two-way ANOVA was conducted with diet and treatment as independent variables. Data from object recognition test expressed as Discrimination Index as described (De Paula et al., 2020), and the difference between groups was assessed by performing an unpaired *t*-test. The differences were considered significant when *p* < 0.05. Outliers were only excluded when a problem was noted during the experiment or sample processing.

## Results

### Short-Term High-Fat Diet Impairs Hippocampal-Dependent Behavioral and Blood–Brain Barrier Permeability

The metabolic profile of mice used in this experimental protocol was previously published in a recent study from our group (Brunetta et al., 2019). The fasting glucose blood levels did not change after 1, 2, or 4 weeks of a HFD intake. However, we found a decrease in glucose uptake from 1 up to 4 weeks compared to SD group, reflecting a glucose intolerance induced by HFD. The impairment of glucose homeostasis was accompanied by higher body mass gain in HFD animals (Brunetta et al., 2019).

We started by investigating the effects of HFD in a time-course manner (1, 2, or 4 weeks) on behavioral tests evaluating learning and memory, as well as mood and motivation (Figure 1A). To evaluate cognitive capacity, mice were subjected to a hippocampal-dependent recognition cued version of the novel object recognition (NOR) test (Figure 1B). Animals fed a SD demonstrated normal recognition memory, as shown by increased exploratory behavior toward the novel object used in the test session, throughout all evaluated periods. However, mice on a HFD did not show an increased discrimination score toward the novel object. This effect was seen at all investigated time-points (1, 2, and 4 weeks). The depressive-like behavior of mice was further tested in the tail suspension test (TST), where immobility time was recorded (Figure 1C). Prior to the TST, the locomotion of all groups was evaluated in the open field test and found to be very similar (Supplementary Figure 1), making any bias due to differences in locomotion unlikely. We found a higher immobility time of animals submitted to HFD compared to the SD group, a difference already observed in the first week of diet consumption which remained increased for the following weeks [*p* = 0.0294 (1 week); *p* = < 0.0001 (2 weeks); *p* = 0.0445 (4 weeks)].

We next examined whether the HFD induced an increase in BBB permeability in the hippocampus of mice. The sodium fluorescein brain permeability assay was used to assess the functionality of the BBB. As observed in Figure 1D, 1 week of HFD significantly increased sodium fluorescein leakage in hippocampus (*p* = 0.0339). Intriguingly, with 2 weeks of HFD, the dye fluorescence concentration was similar to the SD-fed group, increasing again after 4 weeks of HFD (*p* = 0.0293). These results demonstrate that the consumption of a hyperlipidic diet, even for a short period, leads to an impairment in BBB function, besides metabolic alterations.

### Late Effect of High-Fat Diet on Hippocampal Mitochondrial Function and Astrocyte Activation

**FIGURE 2:**
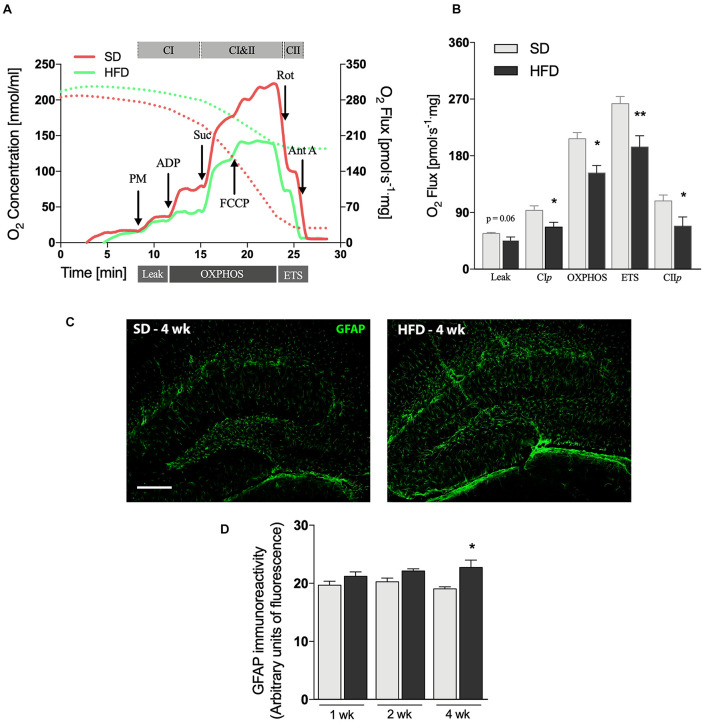
Hippocampal alterations followed by 4 weeks of HFD. **(A)** Representative experiment in SUIT protocol. **(B)** Respirometric measurements by oxygen flux using PM as initial substrates (leak), followed by ADP (CI*p*), S (Oxphos), FCCP titrations (ETS), and finally inhibited by Rot (CII*p*; *F* = 2.077) and Ant A in the hippocampus of mice submitted to 4 weeks of SD or HFD (*n* = 5–6). **(C)** Representative images of GFAP immunolabeling in the hippocampus of 4 weeks SD and HFD-fed mice (scale bar = 150 μm) along with **(D)** quantification of GFAP immunoreactivity (*n* = 4). Values are mean ± SEM. Unpaired *t*-test performed. *p < 0.05, **p < 0.01 SD vs. HFD.

The mitochondrial activity was examined in hippocampi homogenates by high resolution respirometry. No significant changes were observed in hippocampal mitochondrial function up to 2 weeks of HFD (Supplementary Figures 2A,B). However, 4 weeks of HFD-feeding caused an intense mitochondrial dysfunction (Figures 2A,B). We used a multi-substrate protocol to measure the O_2_ consumption rate related to proton leak (LEAK, PM), complex I linked OXPHOS capacity (CI*p*, PM + ADP) and oxidative phosphorylation (OXPHOS, PM + ADP + S) in hippocampal homogenates (Figure 2A). LEAK stimulation with PM, in the non-phosphorylating state, showed a significant lower O_2_ consumption upon 4 weeks of HFD intake (Figure 2B, *p* = 0.063). The addition of succinate (S) to CI*p* stimulated the respiratory OXPHOS capacity, however, this stimulation was 25% lower in animals submitted to a HFD (*p* = 0.0173). To obtain a measure for ETS, i.e., the maximal capacity of the ETS, a step-by-step titration with the uncoupler FCCCP was performed. Our data reveal that 4 weeks of HFD-feeding caused a significant reduction of ETS hippocampal capacity (*p* = 0.0083). The inhibitory effect of rotenone (Rot) on Complex I permits the evaluation of the complex II linked OXPHOS capacity (CII*p*), showing a 60% more activity on SD-fed mice, compared to HFD-fed mice (Figure 4B, *p* = 0.0393).

Astrogliosis is a hallmark of CNS injury and has been observed in several mouse models of neurodegeneration by using immunofluorescence analysis (Brenner, 2014). GFAP labeling (Figures 2C,D) was significantly increased in the hippocampus of mice fed HFD for 4 weeks (*p* = 0.0319), whereas the modest increases observed at the earlier time-points did not reach statistical significance (Supplementary Figure 2C).

### The Early Time-Course of High-Fat Diet-Induced Brain Impairments

**FIGURE 3:**
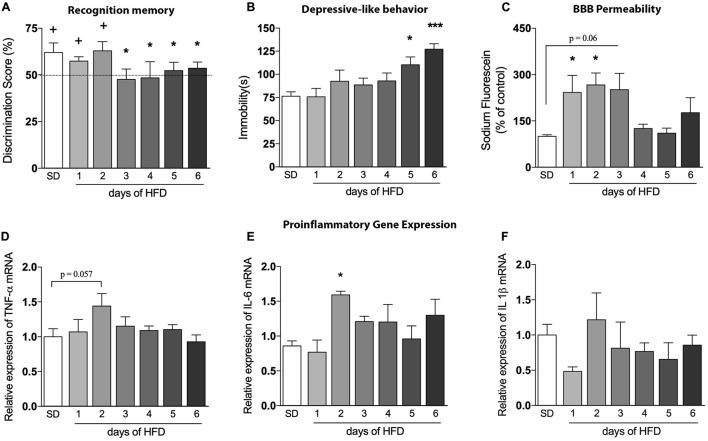
Evaluation of the high-fat diet effects in an early time-course. Assessment of the onset changes in **(A)** cognition (*n* = 6–8/group; one-sample *t*-test with hypothetical value of 50%), **(B)** depressive-like behavior (*n* = 6–8), **(C)** BBB leakage in hippocampus of mice fed HFD from 1 up to 6 days (*n* = 4), as well as proinflammatory cytokines represented by **(D)** TNF-α, **(E)** IL-6, and **(F)** IL-1β (*n* = 4). One-way ANOVA followed by Dunnett *post hoc* test performed for **(B–F)**. *p < 0.05, ***p < 0.001 SD vs. HFD. Values are mean ± SEM. ^+^*p* < 0.05 vs. chance levels (50% of a new object investigation in test trial).

To determine the exact onset of brain and behavioral alterations, we evaluated the parameters described above daily during the first 6 days of HFD exposure. No changes on animals’ body weight were observed during this short period of HFD consumption (data not shown). Figure 3A shows deficits in cognition after only 3 days of HFD, while the depressive-like phenotype can be visualized after day 5 of such diet [Figure 3B, *p* = 0.0385 (day 5); *p* = 0.0007 (day 6)]. The BBB permeability of HFD-fed mice was significantly increased in the hippocampus after 1 (Figure 3C, *p* = 0.0418) and 2 days (*p* = 0.0215), with a similar trend on the third day (*p* = 0.0610). The fluorescein dye leakage was normalized at day 4, returning to basal levels.

**FIGURE 4:**
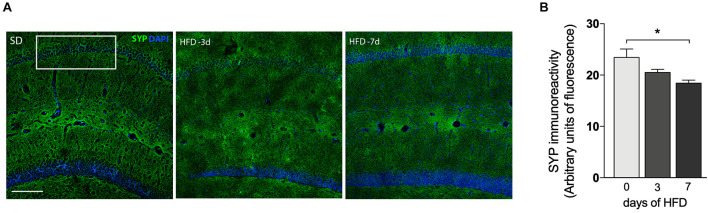
Decrease of synaptic density after 7 days of HFD. **(A)** Immunofluorescence analysis for the expression and localization of the pre-synaptic terminals immunolabeled for pre-synaptic marker synaptophysin (SYP) in hippocampal CA1 region of mice submitted to SD (0), 3 or 7 days of HFD (*n* = 4–5, one-way ANOVA followed by Dunnett *post hoc* test). SYP represented in green and DAPI represented in blue. Scale bar = 50 μm **(B)** quantitative analysis for the SYP immunoreactivity. Data are shown as mean ± SEM. *p < 0.05 SD vs. HFD.

**FIGURE 5:**
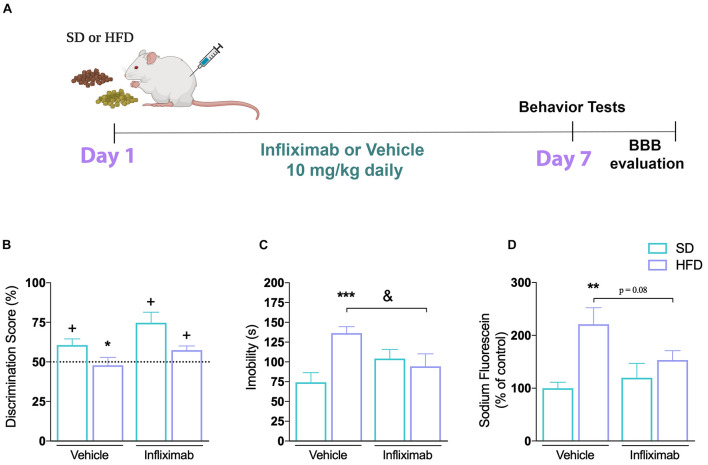
Anti-inflammatory effect on CNS alterations induced by high-fat diet. **(A)** Experimental design of mice fed either SD or HFD for 7 days and treated with saline or infliximab (10 mg/kg). **(B)** Cognitive performance of treated mice (*n* = 7–8/group; one-sample *t*-tests). **(C)** Time of immobility in TST (*n* = 7–8; two-way ANOVA followed by Duncan *post hoc* test). **(D)** Hippocampal sodium fluorescein assay (*n* = 4/group; two-way ANOVA followed by Duncan *post hoc* test). Values are mean ± SEM. ^+^*p* < 0.05 vs. chance levels (50% of a new object investigation in test trial). **p < 0.01 compared with mice fed with SD and ^&^*p* < 0.05 compared HFD mice treated with vehicle. *p < 0.05, **p < 0.01, ***p < 0.001 SD vs. HFD.

In addition, we sought to explore inflammatory markers in the hippocampus of HFD-fed mice to evaluate a possible connection between neuroinflammation, BBB dysfunction, and behavioral outcomes. The mRNA levels of TNF-α was slightly induced on the second day after HFD introduction (Figure 3D, *p* = 0.0572), but returned to baseline levels thereafter. Likewise, a significant increase of IL-6 was also observed (Figure 3E, *p* = 0.0154), with a similar return to baseline levels in the following days. No significant differences in IL-1β mRNA levels were observed during the 6 days (Figure 3F).

We also investigated whether mice consuming HFD for up to 7 days would exhibit alterations in synaptic density. Immunofluorescence staining for the presynaptic marker SYP was conducted in the hippocampal CA1 field (Figure 4A) to visualize presynaptic terminals. The intensity of SYP was analyzed by immunostaining which displayed a 25.7% decrease (Figure 4B, *p* = 0.0079) in mice submitted to 7 days of HFD compared to the control group. No significant differences were detected under 3 days of HFD feeding.

### Immunoneutralization of TNF-α Modulated Behavior and Blood–Brain Barrier Impairments

In clinical practice, the inhibition of TNF-α activity has proven to have beneficial effects in chronic inflammatory and metabolic diseases (Araújo et al., 2007). We used a TNF-α inhibitor to determine if TNF-α was critical for the behavioral impairments and the BBB dysfunction seen in response to HFD. We evaluated the effect of the TNF-α blocking monoclonal antibody Infliximab in mice submitted to SD or HFD, as shown in Figure 5A. Our results show that the impaired recognition memory observed in mice after 1 week of HFD consumption was mitigated by the cotreatment with Infliximab (Figure 5B). Thus, HFD-treated mice given vehicle did not show any preference toward the novel object, presenting a discrimination score similar to the random chance, whereas a better performance was observed on HFD-treated mice given Infliximab. The mice exposed to the HFD diet treated with Infliximab explored for a longer time the new object. Furthermore, the two-way ANOVA revealed a significant effect for diet and treatment interaction on mice depression phenotype analysis [*F*(1,34) = 8.75, *p* < 0.01] performed in the TST. Subsequent Duncan’s *post hoc* test pointed out that the immunoneutralization of TNF-α mediated by Infliximab treatment restored the HFD diet-induced depression phenotype in mice (Figure 5C). In addition, the two-way ANOVA indicated that diet and Infliximab treatment tend to interact on fluorescein permeability [*F*(1,12) = 4.25, *p* = 0.06] in the hippocampus of mice. Subsequent *post hoc* comparisons demonstrated an increased sodium fluorescein permeability in the hippocampus of mice exposed to HFD, which was trend to be reduced by TNF-α inhibition (Figure 5D).

## Discussion

Clinical evidence show that only 4-day consumption of a Western-style diet impacts hippocampal-dependent learning and memory in young adults (Attuquayefio et al., 2017). Studies in rodents receiving a HFD have corroborated this evidence, showing impairments especially in spatial tasks in which the hippocampus is required (Campbell and MacQueen, 2004; McNay et al., 2010; Toups et al., 2013; Mansur et al., 2015). However, the temporal window in which these effects take place, and the molecular mechanism eliciting the hippocampal dysfunction are currently unclear. This study reveals not only that a short exposure to HFD impacts brain function, but also the timing of these changes occur over time. Our figures results are represented as the time-course in which alterations occur. We highlight, in a chronological manner, the key events involved on behavioral changes caused by HFD. Our outcomes point to a rapid deficit in hippocampal and emotional dependent tasks in mice fed HFD, which was concomitant with the induction of proinflammatory cytokines and BBB permeability in the hippocampus. Furthermore, we also showed that by inhibiting the TNF-α pathway, behavioral changes are mitigated. The use of an already approved drug, like Infliximab, improve the translational features of the work.

Studies that describe the effects of a short exposure to HFD (up to 4 weeks) on learning and memory, as well as on mood behavior appear to vary remarkably due to diversity among animal species and strains, diets composition, the length of exposure, and the method used to assess behavioral outcomes. Cognitive impairment was seen in juvenile mice fed with a 60% HFD for 1 or 3 weeks (Kaczmarczyk et al., 2013), whereas we found an impaired memory function already 3 days after the start of a HFD intake. In aged rats, fear memory was impaired after a 3-day-HFD but remained intact in young adults (Spencer et al., 2017). Using multiple types of associative memory tasks, it was reported a rapid deficit in episodic, spatial, and context memories, reversing to normal parameters when switching back to a low-fat diet (McLean et al., 2018). Another time-course study has demonstrated HFD-inducing memory deficits after longer periods of feeding (Saiyasit et al., 2020).

Among the neuropathologies associated with obesity, perhaps the most frequent is related to mood disorders. Experimental mouse models of depression and clinical studies have described structural changes in the hippocampus (Campbell and MacQueen, 2004; Vagena et al., 2019). Our findings presented here are consistent with previous studies on the interconnectedness of depression and obesity (Sharma et al., 2010; Abildgaard et al., 2011; MacQueen and Frodl, 2011; Toups et al., 2013; Mansur et al., 2015; Vagena et al., 2019; Lama et al., 2020). We observed depression-like behavior, in the TST, after only 5 days of a HFD in mice. In this regard, preclinical evidence indicates that either 3 weeks dietary or genetically induced obesity in mice lead to a depressive phenotype observed in both the TST and the forced swim test (Vagena et al., 2019). Similar findings have also been reported in an animal model of T2D (Sharma et al., 2010). Moreover, a study using the Flinders Sensitive Line rat, a validated genetic animal model of depression, found that HFD exacerbated depressive-like features, despite not affecting non-depressed rats (Abildgaard et al., 2011). In a recent study using a wide battery of behavioral tests, it was reported that 12 weeks of HFD causes a depressive-like phenotype including anhedonia, one of the key symptoms of depression (Lama et al., 2020).

An intriguing question raised by these data is which specific events that trigger the HFD-induced behavioral changes? Several studies report the consequences of HFD on BBB permeability (Stranahan et al., 2016; Guillemot-Legris and Muccioli, 2017; Ramalho et al., 2018). For instance, BBB leakage was implicated as a contributing factor to obesity-induced neuroinflammation and cognitive deficits in a genetic model of obesity (Stranahan et al., 2016). Here we show an impaired BBB functionality already on the first day of HFD prematurely affecting the behavioral outcomes. The plasticity and dynamics of the BBB continually adjust to maintain homeostasis, either by adapting to changes in different physiological states or by adapting to the exchange of molecules between the bloodstream and the brain (Segarra et al., 2021). This may explain why the initial response to the introduction of HFD has temporarily disappeared, oscillating between greater permeability and a return to baseline conditions. The present data is in accordance with a previous study showing that the hypothalamic median eminence and surrounding areas got more permeable to peripheral signals after 1 week of HFD introduction, followed by a return to normal conditions at 2 weeks and a second increase in permeability on the fourth week of HFD (Ramalho et al., 2018).

Experimental studies have sought to identify the mechanisms triggering the neuronal modulation resulting from increased BBB permeability in diet-induced obesity. Among them, inflammation (De Souza et al., 2005; Pistell et al., 2010; Thaler et al., 2012; Spencer et al., 2017) and mitochondrial abnormalities (Dietrich et al., 2013; Carraro et al., 2018) stand out. However, it is still unknown which of these events is the primary trigger. Rodent models of diet-induced obesity are characterized by inflammation in both peripheral tissues and CNS areas critical to energy homeostasis (De Souza et al., 2005; Thaler et al., 2012; Dietrich et al., 2013; Stranahan et al., 2016; Nakandakari et al., 2019). The study by Thaler et al. (2012) was a pioneer in demonstrating the increase of inflammatory cytokines in the hypothalamus much earlier (1–3 days) than in peripheral tissues (4 weeks) when animals are subjected to HFD (60% of calories from fat) (Thaler et al., 2012). Interestingly, as in our data, the study shows an oscillating expression of a range of inflammatory genes. Modulation of hippocampal pro-inflammatory (TNF-α and IL-1β) and apoptotic (Bcl2 and BAX) genes were recently observed in mice after 3 days of HFD (Nakandakari et al., 2019). Also, the short-term HFD was enough to increase the hippocampal IL-1β content in aged rats (Spencer et al., 2017).

Here we observed an increase in hippocampal IL-6 and a trend (*p* = 0.057) of increase in TNF-α gene expression (from the second day of HFD), even before the metabolic changes could be detected (Brunetta et al., 2019). These findings indicate that an inflammatory process occurs quickly after HFD exposure and may be involved in the early stages of hippocampal dysfunction. However, we noted that the increase in GFAP only after 4 weeks of HFD, suggesting that the astrocyte activation is involved in the progression, rather than the initiation, of hippocampal dysfunction. Importantly our findings suggest that, by inhibiting one of the inflammatory pathways, the behavioral impairments and BBB dysfunction are mitigated. As a proof of concept, upon neutralization of TNF-α, dysfunctions in BBB permeability and cognition, as well as depressive-like phenotype induced by HFD were no longer observed. Indeed, the peripheral administration of the anti-TNF-α Infliximab has previously been shown to restore glucose homeostasis of 8 weeks HFD-induced obesity and diabetes (Araújo et al., 2007). After high-fat intake, the reduction of peripheral and central inflammation with Infliximab was associated with a decrease in the hypothalamic expression not only of TNF-α but also of other cytokines, such as IL-6 and IL-1β (Milanski et al., 2012). Furthermore, Infliximab either prevented the impairment of spatial memory in rats with hepatic encephalopathy (Dadsetan et al., 2016), however, there were no reports that demonstrated its role in mitigating cognitive deficits induced by the consumption of saturated fat.

The hippocampus is particularly prone to develop deficits in synaptic transmission and density during inflammatory processes and is also sensitive to the damaging effect of pro-inflammatory cytokines (Hauss-Wegrzyniak et al., 2002; Costello et al., 2011). The reduction in the hippocampal CA1 staining of presynaptic protein observed here suggests that changes in synaptic density occurs earlier than previously reported. Liu et al. (2015) and Hwang et al. (2010) reported changes in synaptophysin levels after 12 weeks and 9–12 months of HFD, respectively. Interestingly, in many neurological disorders, inflammation, neuronal degeneration and synaptic dysfunction coexist (Di Filippo et al., 2008, 2013).

Experimental studies have shown that dietary fats can disturb mitochondria dynamics in hypothalamic neurons and directly affect energy homeostasis (Dietrich et al., 2013; Carraro et al., 2018). We hypothesized that mitochondrial dysfunction could trigger the behavioral outcomes; however, we observed alterations in hippocampal mitochondrial oxygen consumption only at the later point, after 4 weeks of HFD. Our data goes in the same direction as to what has been described in the hypothalamus. The consumption of high-fat for 16 weeks modifies the morphology and density of hypothalamic mitochondria in AgRP^–/–^ mice (Dietrich et al., 2013), while no changes in hypothalamic mitochondrial respiration were observed after 1 or 7 days of HFD (Carraro et al., 2018). It seems that the hippocampal mitochondria initially can resist the challenge of inflammation and increased BBB permeability. However, prolonged exposure to saturated fats results in hippocampal mitochondrial dysfunction.

In conclusion, our observations indicate that overconsumption of dietary fats cause a dynamic and rapid hippocampal impairment. As summarized in the graphical abstract, HFD rapidly brings up deficits in memory and mood behavior. These events are associated with an early increase in hippocampal BBB permeability and inflammatory cytokine production, culminating in synaptic dysfunction. The later impairment in mitochondrial function and astrocyte activation could indicate a role in the progression of the hippocampal dysfunction. We acknowledge that studding part of neuroinflammation pathway and not specifying inflammatory and synaptic protein markers in hippocampus under different approaches is a limitation of this study that could be further explored in the future.

## Data Availability Statement

The raw data supporting the conclusions of this article will be made available by the authors, without undue reservation.

## Ethics Statement

The animal study was reviewed and approved by the Federal University of Santa Catarina’s Ethical Review Committee for Animal Experimentation (Protocol number 6191300316).

## Author Contributions

AB and JO: conceptualization. GP: methodology, data analysis, and writing original draft. HB: mitochondrial and metabolic assays. DFE and JG: formal analysis. AB, DE, and LV: funding acquisition. DE: writing – review and editing. All authors contributed to the article and approved the submitted version.

## Conflict of Interest

The authors declare that the research was conducted in the absence of any commercial or financial relationships that could be construed as a potential conflict of interest.

## Publisher’s Note

All claims expressed in this article are solely those of the authors and do not necessarily represent those of their affiliated organizations, or those of the publisher, the editors and the reviewers. Any product that may be evaluated in this article, or claim that may be made by its manufacturer, is not guaranteed or endorsed by the publisher.

## References

[B1] Abildgaard A. Solskov L. Volke V. Harvey B. H. Lund S. Wegener G. (2011). A high-fat diet exacerbates depressive-like behavior in the flinders sensitive line (FSL) rat, a genetic model of depression. *Psychoneuroendocrinology* 36 623–633. 10.1016/j.psyneuen.2010.09.004 20888697

[B2] Araújo E. P. De Souza C. T. Ueno M. Cintra D. E. Bertolo M. B. Carvalheira J. B. (2007). Infliximab restores glucose homeostasis in an animal model of diet-induced obesity and diabetes. *Endocrinology* 148 5991–5997. 10.1210/en.2007-0132 17761768

[B3] Arroyo-Johnson C. Mincey K. D. (2016). Obesity epidemiology worldwide. *Gastroenterol. Clin. North Am.* 45 571–579. 10.1016/j.gtc.2016.07.012 27837773 PMC5599163

[B4] Asfaw A. (2011). Does consumption of processed foods explain disparities in the body weight of individuals? The case of guatemala. *Health Econ.* 20 184–195. 10.1002/hec.1579 20029821

[B5] Attuquayefio T. N. Stevenson R. J. Oaten M. J. Francis H. M. (2017). A four-day Western-style dietary intervention causes reductions in hippocampal-dependent learning and memory and interoceptive sensitivity. *PLoS One* 12:e0172645. 10.1371/journal.pone.0172645 28231304 PMC5322971

[B6] Baym C. L. Naiman A. K. Monti J. M. Raine L. B. Drollette E. S. Moore R. D. (2014). Dietary lipids are dif- ferentially associated with hippocampal dependent relational memory in prepubescent children. *Am. J. Clin. Nutr.* 99 1026–1032. 10.3945/ajcn.113.079624 24522447 PMC3985209

[B7] Beilharz J. E. Maniam J. Morris M. J. (2014). Short exposure to a diet rich in both fat and sugar or sugar alone impairs place, but not object recognition memory in rats. *Brain Behav. Immun.* 37 134–141. 10.1016/j.bbi.2013.11.016 24309633

[B8] Brenner M. (2014). Role of GFAP in CNS injuries. *Neurosci. Lett.* 17 7–13. 10.1016/j.neulet.2014.01.055 24508671 PMC4049287

[B9] Brunetta H. S. de Paula G. C. de Oliveira J. Martins E. L. Dos Santos G. J. Galina A. (2019). Decrement in resting and insulin-stimulated soleus muscle mitochondrial respiration is an early event in diet-induced obesity in mice. *Exp. Physiol.* 104 306–321. 10.1113/EP087317 30578638

[B10] Campbell S. MacQueen G. (2004). The role of the hippocampus in the pathophysiology of major depression. *J. Psychiatry Neurosci.* 29 417–426. 15644983 PMC524959

[B11] Carraro R. S. Souza G. F. Solon C. Razolli D. S. Chausse B. Barbizan R. (2018). Hypothalamic mitochondrial abnormalities occur downstream of inflammation in diet-induced obesity. *Mol. Cell Endocrinol.* 460 238–245. 10.1016/j.mce.2017.07.029 28760600

[B12] Cohen S. J. Stackman R. W. Jr. (2015). Assessing rodent hippocampal involvement in the novel object recognition task. A review. *Behav. Brain Res.* 285 105–117. 10.1016/j.bbr.2014.08.002 25169255 PMC7008635

[B13] Costello D. A. Watson M. B. Cowley T. R. Murphy N. Murphy Royal C. Garlanda C. (2011). Interleukin-1alpha and HMGB1 mediate hippocampal dysfunction in SIGIRR-deficient mice. *J. Neurosci.* 31 3871–3879. 10.1523/JNEUROSCI.6676-10.2011 21389242 PMC6622806

[B14] Dadsetan S. Balzano T. Forteza J. Cabrera-Pastor A. Taoro-Gonzalez L. Hernandez-Rabaza V. (2016). Reducing peripheral inflammation with infliximab reduces neuroinflammation and improves cognition in rats with hepatic encephalopathy. *Front. Mol. Neurosci.* 9:106. 10.3389/fnmol.2016.00106 27853420 PMC5089983

[B15] Davidson T. L. Hargrave S. L. Swithers S. E. Sample C. H. Fu X. Kinzig K. P. (2013). Inter-relationships among diet, obesity and hippocampal-dependent cognitive function. *Neuroscience* 253 110–122. 10.1016/j.neuroscience.2013.08.044 23999121 PMC3934926

[B16] Davidson T. L. Kanoski S. E. Schier L. A. Clegg D. J. Benoit S. C. (2007). A potential role for the hippocampus in energy intake and body weight regulation. *Curr. Opin. Pharmacol.* 7 613–616. 10.1016/j.coph.2007.10.008 18032108 PMC2223183

[B17] de Oliveira J. Engel D. F. de Paula G. C. Dos Santos D. B. Lopes J. B. Farina M. (2020). High cholesterol diet exacerbates blood-brain barrier disruption in LDLr-/- mice: impact on cognitive function. *J. Alzheimers Dis.* 78 97–115. 10.3233/JAD-200541 32925052 PMC7683087

[B18] De Paula G. C. de Oliveira J. Engel D. F. Lopes S. C. Moreira E. L. G. Figueiredo C. P. (2020). Red wine consumption mitigates the cognitive impairments in low-density lipoprotein receptor knockout (LDLr-/-) mice. *Nutr. Neurosci.* 10 1–11. 10.1080/1028415X.2019.1704472 31910791

[B19] De Souza C. T. Araujo E. P. Bordin S. Ashimine R. Zollner R. L. Boschero A. C. (2005). Consumption of a fat-rich diet activates a proinflammatory response and induces insulin resistance in the hypothalamus. *Endocrinology* 146 4192–4199. 10.1210/en.2004-1520 16002529

[B20] Di Filippo M. Chiasserini D. Gardoni F. Viviani B. Tozzi A. Giampà C. (2013). Effects of central and peripheral inflammation on hippocampal synaptic plasticity. *Neurobiol. Dis.* 52 229–236. 10.1016/j.nbd.2012.12.009 23295855

[B21] Di Filippo M. Sarchielli P. Picconi B. Calabresi P. (2008). Neuroinflammation and synaptic plasticity: theoretical basis for a novel, immune-centred, therapeutic approach to neurological disorders. *Trends Pharmacol. Sci.* 29 402–412. 10.1016/j.tips.2008.06.005 18617277

[B22] Dietrich M. O. Liu Z. W. Horvath T. L. (2013). Mitochondrial dynamics controlled by mitofusins regulate agrp neuronal activity and diet-induced obesity. *Cell* 155 188–199. 10.1016/j.cell.2013.09.004 24074868 PMC4142434

[B23] Francis H. M. Stevenson R. J. (2011). Higher reported saturated fat and refined sugar intake is associated with reduced hippocampal-dependent memory and sensitivity to interoceptive signals. *Behav. Neurosci.* 125 943–955. 10.1037/a0025998 22023100

[B24] Freeman L. R. Haley-Zitlin V. Rosenberger D. S. Granholm A. C. (2014). Damaging effects of a high-fat diet to the brain and cognition: a review of proposed mechanisms. *Nutr. Neurosci.* 17 241–251. 10.1179/1476830513Y.0000000092 24192577 PMC4074256

[B25] Gibson E. L. Barr S. Jeanes Y. M. (2013). Habitual fat intake predicts memory function in younger women. *Front. Hum. Neurosci.* 7:838. 10.3389/fnhum.2013.00838 24376410 PMC3858814

[B26] Guillemot-Legris O. Muccioli G. G. (2017). Obesity-induced neuroinflammation: beyond the hypothalamus. *Trends Neurosci.* 40 237–253. 10.1016/j.tins.2017.02.005 28318543

[B27] Hao S. Dey A. Yu X. Stranahan A. M. (2016). Dietary obesity reversibly induces synaptic stripping by microglia and impairs hippocampal plasticity. *Brain Behav. Immun.* 51 230–239. 10.1016/j.bbi.2015.08.023 26336035 PMC4679537

[B28] Hauss-Wegrzyniak B. Lynch M. A. Vraniak P. D. Wenk G. L. (2002). Chronic brain inflammation results in cell loss in the entorhinal cortex and impaired LTP in perforant path-granule cell synapses. *Exp. Neurol.* 176 336–341. 10.1006/exnr.2002.7966 12359175

[B29] Hwang L. L. Wang C. H. Li T. L. Chang S. D. Lin L. C. Chen C. P. (2010). Sex differences in high-fat diet-induced obesity, metabolic alterations and learning, and synaptic plasticity deficits in mice. *Obesity* 18 463–469. 10.1038/oby.2009.273 19730425

[B30] Kaczmarczyk M. M. Machaj A. S. Chiu G. S. Lawson M. A. Gainey S. J. York J. M. (2013). Methylphenidate prevents high-fat diet (HFD)-induced learning/memory impairment in juvenile mice. *Psychoneuroendocrinology* 38 1553–1564. 10.1016/j.psyneuen.2013.01.004 23411461 PMC3659210

[B31] Kanoski S. E. Davidson T. L. (2011). Western diet consumption and cognitive impairment: links to hippocampal dysfunction and obesity. *Physiol. Behav.* 103 59–68. 10.1016/j.physbeh.2010.12.003 21167850 PMC3056912

[B32] Kanoski S. E. Zhang Y. Zheng W. Davidson T. L. (2010). The effects of a high-energy diet on hippocampal function and blood-brain barrier integrity in the rat. *J. Alzheimers Dis.* 21 207–219. 10.3233/JAD-2010-091414 20413889 PMC4975946

[B33] Kratz M. Baars T. Guyenet S. (2013). The relationship between high-fat dairy consumption and obesity, cardiovascular, and metabolic disease. *Eur. J. Nutr.* 52 1–24. 10.1007/s00394-012-0418-1 22810464

[B34] Lama A. Pirozzi C. Annunziata C. Morgese M. G. Senzacqua M. Severi I. (2020). Palmitoylethanolamide counteracts brain fog improving depressive-like behaviour in obese mice: possible role of synaptic plasticity and neurogenesis. *Br. J. Pharmacol.* 178 845–859. 10.1111/bph.15071 32346865

[B35] Liu Z. Patil I. Y. Jiang T. Sancheti H. Walsh J. P. Stiles B. L. (2015). High-fat diet induces hepatic insulin resistance and impairment of synaptic plasticity. *PLoS One* 10:e0128274. 10.1371/journal.pone.0128274 26023930 PMC4449222

[B36] MacQueen G. Frodl T. (2011). The hippocampus in major depression: evidence for the convergence of the bench and bedside in psychiatric research? *Mol. Psychiatry* 16 252–264. 10.1038/mp.2010.80 20661246

[B37] Makrecka-Kuka M. Krumschnabel G. Gnaiger E. (2015). High-resolution respirometry for simultaneous measurement of oxygen and hydrogen peroxide fluxes in permeabilized cells, tissue homogenate and isolated mitochondria. *Biomolecules* 5 1319–1338. 10.3390/biom5031319 26131977 PMC4598754

[B38] Mansur R. B. Brietzke E. McIntyre R. S. (2015). Is there a “metabolic-mood syndrome”? A review of the relationship between obesity and mood disorders. *Neurosci. Biobehav. Rev.* 52 89–104. 10.1016/j.neubiorev.2014.12.017 25579847

[B39] McLean F. H. Grant C. Morris A. C. Horgan G. W. Polanski A. J. Allan K. (2018). Rapid and reversible impairment of episodic memory by a high-fat diet in mice. *Sci. Rep.* 8:11976. 10.1038/s41598-018-30265-4 30097632 PMC6086894

[B40] McNay E. C. Ong C. T. McCrimmon R. J. Cresswell J. Bogan J. S. Sherwin R. S. (2010). Hippocampal memory processes are modulated by insulin and high-fat-induced insulin resistance. *Neurobiol. Learn. Mem.* 93 546–553. 10.1016/j.nlm.2010.02.002 20176121 PMC2878207

[B41] Milanski M. Arruda A. P. Coope A. Ignacio-Souza L. M. Nunez C. E. Roman E. A. (2012). Inhibition of hypothalamic inflammation reverses diet-induced insulin resistance in the liver. *Diabetes* 61 1455–1462. 10.2337/db11-0390 22522614 PMC3357298

[B42] Morris M. C. Evans D. A. Tangney C. C. Bienias J. L. Schneider J. A. Wilson R. S. (2006). Dietary copper and high saturated and trans fat intakes associated with cognitive decline. *Arch. Neurol.* 63 1085–1088. 10.1001/archneur.63.8.1085 16908733

[B43] Nakandakari S. C. B. R. Muñoz V. R. Kuga G. K. Gaspar R. C. Sant’Ana M. R. Pavan I. C. B. (2019). Short-term high-fat diet modulates several inflammatory, ER stress, and apoptosis markers in the hippocampus of young mice. *Brain Behav. Immun.* 79 284–293. 10.1016/j.bbi.2019.02.016 30797044

[B44] Pistell P. J. Morrison C. D. Gupta S. Knight A. G. Keller J. N. Ingram D. K. (2010). Cognitive impairment following high fat diet consumption is associated with brain inflammation. *J. Neuroimmunol.* 219 25–32. 10.1016/j.jneuroim.2009.11.010 20004026 PMC2823983

[B45] Ramalho A. F. Bombassaro B. Dragano N. R. Solon C. Morari J. Fioravante M. (2018). Dietary fats promote functional and structural changes in the median eminence blood/spinal fluid interface-the protective role for BDNF. *J. Neuroinflammation* 15:10. 10.1186/s12974-017-1046-8 29316939 PMC5761204

[B46] Reardon T. Timmer C. P. Barret C. B. Berdegue J. A. (2003). The rise of supermarkets in Africa, Asia, and Latin America. *Am. J. Agric. Econ.* 85 1140–1146. 10.1111/j.0092-5853.2003.00520.x

[B47] Ross A. P. Bartness T. J. Mielke J. G. Parent M. B. (2009). A high fructose diet impairs spatial memory in male rats. *Neurobiol. Learn. Mem.* 92 410–416. 10.1016/j.nlm.2009.05.007 19500683 PMC2737072

[B48] Saiyasit N. Chunchai T. Prus D. Suparan K. Pittayapong P. Apaijai N. (2020). Gut dysbiosis develops prior to metabolic disturbance and cognitive decline in high-fat-diet induced obese condition. *Nutrition* 69:110576. 10.1016/j.nut.2019.110576 31580986

[B49] Segarra M. Aburto M. R. Acker-Palmer A. (2021). Blood-brain barrier dynamics to maintain brain homeostasis. *Trends Neurosci.* 44 393–405. 10.1016/j.tins.2020.12.002 33423792

[B50] Sharma A. N. Elased K. M. Garrett T. L. Lucot J. B. (2010). Neurobehavioral deficits in db/db diabetic mice. *Physiol. Behav.* 101 381–388. 10.1016/j.physbeh.2010.07.002 20637218 PMC3098504

[B51] Sims N. R. Blass J. P. (1986). Expression of classical mitochondria1 respiratory responses in homogenates of rat forebrain. *J. Neurochem.* 47 496–505. 10.1111/j.1471-4159.1986.tb04529.x 3734792

[B52] Spencer S. J. D’Angelo H. Soch A. Watkins L. R. Maier S. F. Barrientos R. M. (2017). High-fat diet and aging interact to produce neuroinflammation and impair hippocampal- and amygdalar-dependent memory. *Neurobiol. Aging* 58 88–101. 10.1016/j.neurobiolaging.2017.06.014 28719855 PMC5581696

[B53] Steru L. Chermat R. Thierry B. Simon P. (1985). The tail suspension test: a new method for screening antidepressants in mice. *Psychopharmacology* 85 367–370. 10.1007/BF00428203 3923523

[B54] Stranahan A. M. Hao S. Dey A. Yu X. Baban B. (2016). Blood–brain barrier breakdown promotes macrophage infiltration and cognitive impairment in leptin receptor-deficient mice. *J. Cereb. Blood Flow Metab.* 36 2108–2121. 10.1177/0271678X16642233 27034250 PMC5363667

[B55] Thaler J. P. Yi C. X. Schur E. A. Guyenet S. J. Hwang B. H. Dietrich M. O. (2012). Obesity is associated with hypothalamic injury in rodents and humans. *J. Clin. Invest.* 122 153–162. 10.1172/JCI59660 22201683 PMC3248304

[B56] Toups M. S. Myers A. K. Wisniewski S. R. Kurian B. Morris D. W. Rush A. J. (2013). Relationship between obesity and depression: characteristics and treatment outcomes with antidepressant medication. *Psychosomal. Med.* 75 863–872. 10.1097/PSY.0000000000000000 24163386 PMC3905462

[B57] Vagena E. Ryu J. K. Baeza-Raja B. Walsh N. M. Syme C. Day J. P. (2019). A high-fat diet promotes depression-like behavior in mice by suppressing hypothalamic PKA signaling. *Transl. Psychiatry* 9:141. 10.1038/s41398-019-0470-1 31076569 PMC6510753

[B58] Velloso L. A. Schwartz M. W. (2011). Altered hypothalamic function in diet-induced obesity. *Int. J. Obes.* 35 1455–1465. 10.1038/ijo.2011.56 21386802 PMC3383790

[B59] Wang Z. Ge Q. Wu Y. Zhang J. Gu Q. Han J. (2020). Impairment of long-term memory by a short-term high-fat diet via hippocampal oxidative stress and alterations in synaptic plasticity. *Neuroscience* 424 24–33. 10.1016/j.neuroscience.2019.10.050 31711814

