# Supplementary material for: Hippocampal Function Is Impaired by a Short-Term High-Fat Diet in Mice: Increased Blood–Brain Barrier Permeability and Neuroinflammation as Triggering Events
Source: Front Neurosci. 2021 Nov 4;15:734158. doi: 10.3389/fnins.2021.734158 (PMC8600238; doi:10.3389/fnins.2021.734158)
Supplement: Supplementary file 1 [file Image_1.pdf]

Supplementary Material

1 Supplementary Figures

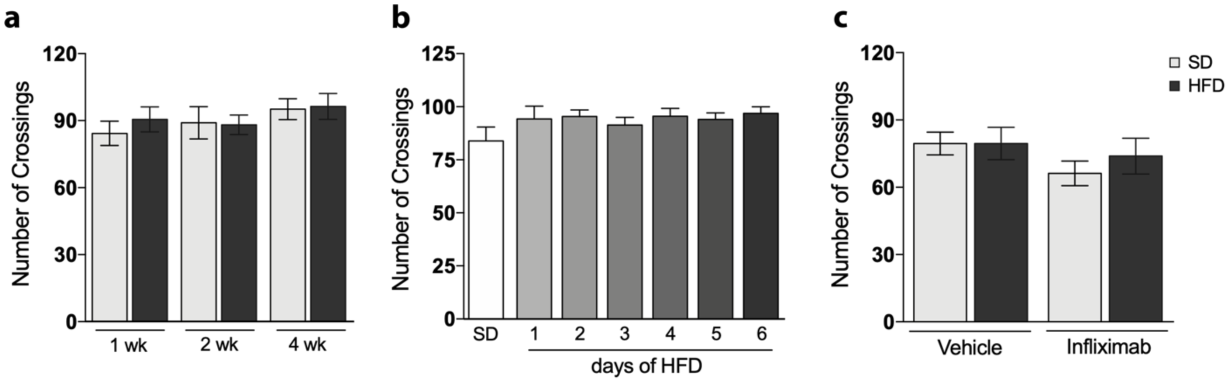

Values are mean ± SEM.

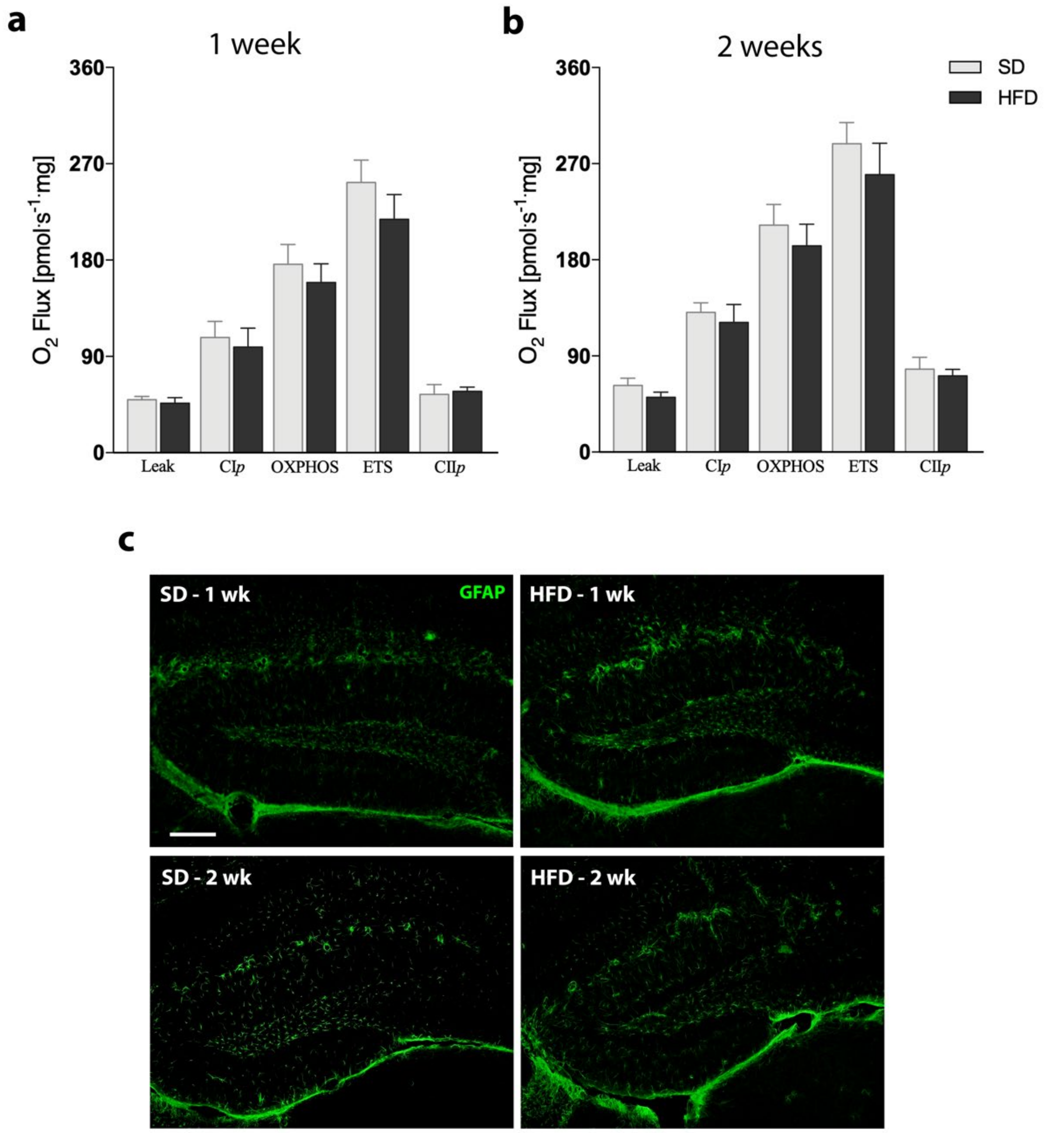

Supplementary Figure 2. Mitochondrial function and astrocyte activation within 1 and 2 weeks of HFD. a-b) Respirometric measurements by oxygen flux in the hippocampus of mice submitted to 1 (a) and 2 (b) weeks of SD or HFD (n= 5-6). **c)** Representative images of GFAP immunolabeling in the hippocampus of 1and 2 weeks SD and HFD-treated mice (n= 4). Scale bar = 150 µm. Values are mean ± SEM.
